# Carcinoma of the Pancreas

**Published:** 1890-07

**Authors:** Nelson G. Richmond

**Affiliations:** Fredonia, N. Y.


### CARCINOMA OF THE PANCREAS.[1]

By NELSON G. RICHMOND, M. D., Fredonia, N. Y.

THE infrequency of Carcinoma of the Pancreas, and the difficulties surrounding a diagnosis, are facts admitted by all. Niemeyer's Text-Book and Hartshorn's Essentials of Practical Medicine have nothing to say on the subject. Flint condenses it into two pages. Ziemssen's Cyclopedia and Pepper's System each discuss it more fully.

I well remember the joke a medical friend of mine perpetrated upon a brother practitioner some years ago. At a post-mortem which was held, the real cause of death could not be discovered. As a way out of the dilemma, a perfectly healthy pancreas was held up and explained as the seat of the disease. The family were perfectly satisfied; so was the other physician. After leaving the house the deception was disclosed and the joke well enjoyed.

The following case of Carcinoma of the Pancreas, recently under observation, may be of interest on account of its rarity:

Mr. B. C., æt. 56, consulted me in February, 1889, with the following history:

The family history was negative, mother dying from a miscarriage, and father living to 87 years.

*Previous History.*—As a boy he was robust and strong, was the champion wrestler, and was nick-named "Stub." When he was thirteen years old, he contracted typhus fever, there being six other cases in the house, two of whom died. The disease was brought from the West by a man whose wife died with it, and whose children were taken after coming East. It became epidemic.

Patient never felt so well after this. While still pale and weak he entered school and was induced to try a "back-hold" with a new boy who had come into the field. With arms around each other's body, the knuckles of his opponent's hands were brought together and so forcibly pushed against the spine as to cause a severe pain, and he dropped to the ground. He "felt something give way," he said, and was never hurt so badly in his life. He said nothing about it at home, but felt weak and sick for some time. Some months later his parents noticed one shoulder drooping. He was taken to a physician, who discovered rotary lateral curvature of the spine.

According to the methods of the time, the treatment was to be either a seton, venesection or — nothing. He was advised to give up farm work and study for a profession, but he remained on the farm. He never had any illness until of late years, but he was peculiarly unlucky as regards accidents, the above being one of a series.

He was at one time tossed up and down on the horns of a short-horn Durham, escaping death almost miraculously, but with bleeding legs and arms, and an injured back. He could not step for six weeks.

Three years ago he slipped on the ice and fell, striking *his back* again on the edge of a wash-tub. It was some time before he could get his breath. At another

---

1. Read at the sixth annual meeting of the Fourth District Branch of New York State Medical Association, held in Buffalo, N. Y., May 13, 1890.

time a horse, grazing, threw up his head suddenly, striking him in the epigastrium. He was knocked back several steps, and hurt badly. He was also run away with by a team, and injured in the head. Even besides these there were a number of minor accidents.

When a boy and young man he was subject to very severe headaches which often lasted for two weeks. No medicine relieved them. They were thought due to the spinal curvature. For years there has been tenderness over the epigastrium, and he hardly knew what it was to be without pain. For the past ten to twelve years he has had bilious colic, morphia alone relieving him. He had these attacks two or three times in a season. His wife noticed failing health for some years. He did not increase in weight as formerly during the winter. He has always been a hard-working, very energetic man.

*Present History and Condition.*—In September, 1889, there passed the patient's bowel what he described as soap. There was a scalding sensation, and he felt as if a hot iron were passed up the rectum. This symptom continued, at intervals of a few days, from September to May. Whenever he ate fats it was worse. Starchy foods controlled it. At one time he was put upon peptonized cod-liver oil, but it did not agree with him.

When I first saw him, in February, he complained of pain in the epigastric region, running through to, and up and down spine. The pain radiated in every direction. The entire region was tender to the touch. He had lost flesh, though his appetite and digestion were good. The pain seemed to have no connection with the latter. The tongue was clean; there was no odor to the breath; no jaundice. The peculiar stools, he described, came liquid from the bowel, and when cool formed solid plates of a golden yellow, and could be handled like wax. They covered the feces, and were not mixed with them.

Examination showed a tumor in the epigastric region, presenting a surface the size of a fist. It extended from the region of the gall-bladder across to the left of the median line. It was impossible to find any division between it and the liver, and it seemed a part of the liver. It was tender on palpation and easily detected in the recumbent posture. There was rotary lateral curvature of the spine. The spine curving to the right in the region of the liver displaced the liver forward and to the left. The possibility of this displacement accounting for the enlargement in front was carefully considered.

A probable diagnosis of cancer of the liver was made at the second visit, and a friend outside of the family told of the hopelessness of the case. Treatment was instituted with a view of increasing assimilation — morphia as occasion required. From that time on he required morphia, and whenever out from under the influence of it, the pain was severe. At times he would seem better, and ride out, but always felt worse afterwards. He went to Buffalo to consult Drs.

Park and Stockton; he was also seen by other medical men, with some of whom the diagnosis was that of a malignant growth, with others non-malignant, while still others preferred not to commit themselves. There were also differences of opinion as to its origin. Malignancy was doubted by some from the fact that failure was so gradual, and was followed by some weeks of seeming improvement.

In July, and just again before death, there were periods of icterus, the urine containing bile also. The tumor did not increase in size. Emaciation progressed, pain increased, appetite failed, and digestion was at last attended with a burning sensation. He died very easily in February, 1890.

Autopsy eighteen hours after death. Rigor mortis well marked. Body extremely emaciated. Icterus apparent in skin and conjunctivæ.

Percussion disclosed no tumor in epigastrium, and its former seeming connection with liver was proven false. Upon entering the abdomen, the stomach and liver were pushed away, and a tumor revealed, about the size of a large fist. It proved to be the enlarged head of the pancreas; the tail was normal in size, but together with the head was breaking down throughout its entire extent into a soft, gray, pultaceous mass. The growth involved for some distance the outer wall of the duodenum, the liver, the spleen, and the lesser curvature of the stomach. It also surrounded the common bile duct, occluding it, hence the jaundice, two attacks of which he had had.

The greatest degree of spinal curvature was directly behind the pancreas, at the first lumbar vertebra. Any irritation of surrounding structures, from displacement of the bodies of vertrebræ, would be felt first in the pancreas. This might have been an exciting cause of the disease; his other injuries also were severe blows upon the spine, and in the epigastrium.

Of the fifty cases I have been able to collect, the following are the most prominent symptoms: emaciation, pain, jaundice, fatty stools, chalky or clay-colored stools, tumor in epigastrium. The pain of this disease is more continuous than in cancer of the pylorus, from which it is often difficult to differentiate it. It also radiates in more directions. Jaundice is a very frequent symptom, and occurs from the cancerous mass surrounding and occluding the ductus choledochus. This duct, opening in common with the main pancreatic duct, is first to be involved and its flow of bile obstructed. It would not be surprising to find jaundice a more common symptom in cancer of the pancreas than in cancer of the liver. One author gives it as found in less than one-third of the cases of cancer of liver; another says it was found in sixty seven out of one hundred and forty-six cases. The emaciation is caused by robbing the system of the fats which the pancreas is accustomed to saponify. It was noted in every case.

This brings us to the question of fatty diarrhea or its absence, and its value as a diagnostic symptom in suspected cases of pancreatic disease. It would seem that disease of this organ to any extent would give stearrhea as a pathognomonic symptom and one invariably present. Dr. Jenner says there is no symptom pathognomonic of cancer of the pancreas, and from the absence of this symptom in a large number of cases, we are forced to the same conclusion. Dr. Richard Bright, in 1832, was the first to connect this symptom with disease of the pancreas. He reported three cases, in which the pancreas was so disorganized that its secreting function must have been almost if not entirely lost. Dr. Lloyd and Dr. Elliotson each have reported cases about this time. Prof. Erb, of Heidelberg, recently presented a patient in his clinic with this disease, having stools of a silvery gray color. Chemical analysis showed that one-half the amount of solids was made up of fats. The microscope revealed ascicular crystals which were soluble in ether. In some of the cases where this symptom is not spoken of, fatty diarrhea may have been overlooked, or there may have been the above condition. Erb considers this an exceptional symptom, and that fat in the stools of the amorphous form was usually found.

Erb, Friedrich, Nothnagel, and Gerhardt have recorded cases with fatty diarrhea, and consider that when fatty crystals are present in excess, there is not only pancreatic disease but also occlusion of the bile-duct. Da Costa in 1858 reported thirty-seven cases, fatty stools being present in only three.

Dr. Reginald Fitz has recently made an exhaustive study of Diseases of the Pancreas. He has collected of hemorrhagic pancreatitis, seventeen cases; pancreatic hemorrhage, sixteen cases; suppurative pancreatitis, twenty-two; gangrene of pancreas, fifteen; disseminated fat necrosis, two. In no case is stearrhea spoken of. In five other instances it was reported absent. In many cases it has been controlled by eliminating all fat from the food, as in the history given. Others have noted cases with this symptom present where all fats and foods convertible into fat have been stopped. A tumor in the region of the pancreas in connection with fatty stools must be considered a strong argument in favor of cancer of the pancreas. And yet, on account of its being recorded absent in a greater proportion of cases, we must agree with Jenner that there is no pathognomonic symptom. It is not always easy to differentiate the tumor, *per se*, from gastric, duodenal, and hepatic tumors.

One point we believe to be true is that carcinomatous tumors of the pancreas are limited to a size not larger than one or both fists. The tumor is more obvious in the recumbent posture, since in the erect posture the liver and stomach come over it. This symptom helps to differentiate it from cancer of the pylorus. A full meal also causes it to disappear.

The duration of this disease is from one to two years.

*Varieties.* — Scirrhus cancer of the head of the pancreas is most common; encephaloid next in order; cylindrical-celled epithelioma occurring least often.

*Treatment.* — As regards medicinal treatment little can be said. It is necessary to control the pain as occasion requires. Symptoms are to be met as they arise. A diet which is most easily digested and assimilated.

Artificial digestives whenever they can be used.

In a monograph now issued in a work entitled Experimental Surgery, by Dr. Nicholas Senn, of Milwaukee, among other points advocated in the surgery of the pancreas, are the following:

1. Complete extirpation of the pancreas is usually followed by death, produced either by traumatism or gangrene of duodenum.

2. Partial excision of pancreas in injury or disease is a feasible and justifiable surgical procedure.

3. Complete obstruction of the pancreatic duct uncomplicated by pathological condition of parenchyma of organ never results in the formation of a cyst.

4. Partial excision is indicated in all cases where disease can be removed completely without sacrificing pancreatic digestion or wounding important adjacent organs. In all operations the pancreatic duct must be maintained.

6. Gradual atrophy of the pancreas from nutritive or degenerative changes is not incompatible with health.

I have for your examination a section of an enlarged lymphatic gland taken from the vicinity of the pancreas. It proves to be cylindrical-celled epithelioma.

---

PROFESSOR ZWIBEER, of the University of Bonn, is a very absent-minded man. He was busily engaged in solving some scientific problem. The servant hastily opened the door of his study, and announced a great family event.

"A little stranger has arrived."

"Eh?"

"It is a little boy."

"Little boy? Well, ask him what he wants."—*Humoristische Blatter.*

